# Assessment of select synthetic cannabinoid receptor agonist bias and selectivity between the type 1 and type 2 cannabinoid receptor

**DOI:** 10.1038/s41598-021-90167-w

**Published:** 2021-05-19

**Authors:** Ayat Zagzoog, Asher L. Brandt, Tallan Black, Eunhyun D. Kim, Riley Burkart, Mikin Patel, Zhiyun Jin, Maria Nikolaeva, Robert B. Laprairie

**Affiliations:** 1 College of Pharmacy and Nutrition, University of Saskatchewan, 3B36, Health Sciences Building, 104 Clinic Place, Saskatoon, SK S7N 5E5 Canada; 2 Health Canada, Ottawa, ON Canada; 3 Department of Pharmacology, College of Medicine, Dalhousie University, Halifax, NS Canada

**Keywords:** Drug safety, Pharmacology

## Abstract

The first synthetic cannabinoid receptor agonists (SCRAs) were designed as tool compounds to study the endocannabinoid system’s two predominant cannabinoid receptors, CB1R and CB2R. Unfortunately, novel SCRAs now represent the most rapidly proliferating novel psychoactive substances (NPS) of abuse globally. Unlike ∆^9^-tetrahydrocannabinol, the CB1R and CB2R partial agonist and the intoxicating constituent of *Cannabis*, many SCRAs characterized to date are full agonists of CB1R. Gaining additional insight into the pharmacological activity of these SCRAs is critical to assess and regulate NPSs as they enter the marketplace. The purpose of this study was to assess select SCRAs recently identified by Canadian police, border service agency, private companies and the illicit market as potential CB1R and CB2R agonists. To this end, fifteen SCRAs were screened for in vitro activity and in silico interactions at CB1R and CB2R. Several SCRAs were identified as being highly biased for cAMP inhibition or βarrestin2 recruitment and receptor subtype selectivity between CB1R and CB2R. The indazole ring and halogen-substituted butyl or pentyl moieties were identified as two structural features that may direct βarrestin2 bias. Two highly-biased SCRAs—JWH-018 2′-napthyl-N-(3-methylbutyl) isomer (biased toward cAMP inhibition) and 4-fluoro MDMB-BINACA (biased toward βarrestin2 recruitment) displayed unique and differential in vivo activity in mice. These data provide initial insight into the correlations between structure, signalling bias, and in vivo activity of the SCRAs.

## Introduction

**Figure 1:**
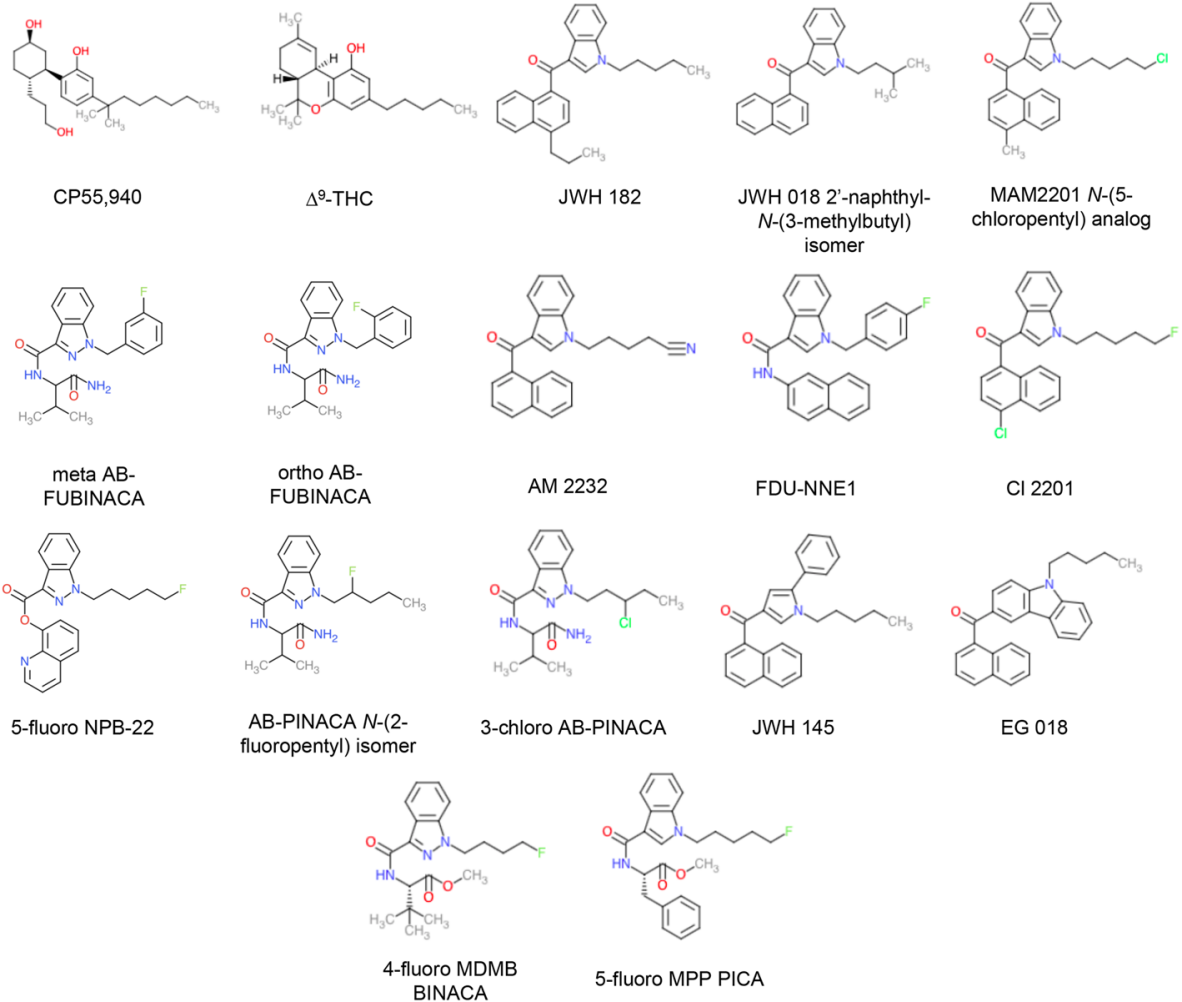
Structures of SCRAs examined in this study. CP55,940 and ∆^9^-THC were used as reference ligands. Chemical structures were drawn in Microsoft PowerPoint by the authors.

In 2018 Canada became the first G7 country to legalize *Cannabis sativa* for medical and recreational purposes. It is critically important that we gain a more comprehensive understanding of cannabinoid pharmacology in order to reduce harm and make full use of this plant’s medical potential. Biologically active compounds in *Cannabis* are referred to as ‘phytocannabinoids’. The two best-known phytocannabinoids are ∆^9^-tetrahydrocannabinol (THC) and cannabidiol (CBD). Our bodies also naturally produce endogenous cannabinoids anandamide (AEA) and 2-arachidonoylglycerol (2-AG). These cannabinoids act to modulate the body’s cannabinoid receptors: CB1R and CB2R, limiting neurotransmitter release throughout the brain and inflammatory processes, respectively ^1^. Beyond these naturally occurring compounds, a wide and structurally diverse array of synthetic cannabinoid receptor agonists (SCRAs) have been produced (Fig. 1) ^1^. These were originally intended to aid in drug development and as tool compounds to help better understand the body’s endogenous cannabinoid system ^1,2^. Unfortunately, many of these compounds are now available through illegal markets where they are sold as psychoactive and intoxicating drugs of misuse known colloquially as ‘spice’ or ‘K-2’^1,2^.

SCRAs pose serious health risks to Canadians as they can lead to severe vomiting, chest pain, increased heart rate, vision blackouts, headaches, kidney damage, agitation, high blood pressure, and psychosis ^3,4^. Acute toxicities of SCRAs such as JWH 018 and JWH 073 are thought to be mediated by CB1R activation ^5–7^; however, novel SCRAs continue to emerge on the market without previous characterization in the scientific literature ^4^. In Canada, the Controlled Drugs and Substances Act (CDSA) provides a legislative framework for the control of substances that can alter mental processes and that may cause harm to the health of an individual or society when misused or diverted to the illegal market. Many SCRAs are controlled under Item 2 of Schedule II to the CDSA under the heading “Synthetic cannabinoid receptor-type one agonists, their salts, derivatives, isomers, and salts of derivatives and isomers”.

The cannabinoid receptors are activated by a wide spectrum of structurally-diverse cannabinoids ^1^. Although classically considered Gα_i/o_-coupled receptors, CB1R and CB2R are a pleiotropically-coupled GPCRs that modulate intracellular signalling through βarrestins and other G proteins ^8–13^. Gα_i/o_-dependent signalling inhibits cAMP accumulation ^8^. In contrast, recruitment of βarrestin1 and βarrestin2 to CB1R results in receptor internalization, recycling or turnover ^11–13^. Ligand bias describes a compound’s ability to promote a certain receptor conformation that favours the preferential coupling with, and signalling through, a specific effector protein relative to other effector proteins ^11^. CB1R and CB2R ligand bias has been observed in multiple cell culture systems and with many SCRAs ^11–13^. Previously, we demonstrated that βarrestin1-biased cannabinoids, such as CP55940 and THC, reduce cellular viability in a cell culture model of Huntington’s disease ^11^. Our data, and the data of other groups ^14–19^, indicate that cannabinoid receptor ligand bias has unique cell-, and potentially organism-level, effects that may explain the unique side effect profiles of SCRAs as compared to *Cannabis*-derived cannabinoids.

SCRAs are one of the most rapidly growing classes of novel psychoactive substances (NPS) and are used as recreational drugs in Canada. Unlike the partial agonist THC, many SCRAs are full agonists of CB1R, which may contribute to these drug’s side effect profile ^1,2^. Recently, several studies have assessed the signaling bias and kinetics of several SCRAs at CB1R through in vitro assays ^14–19^. These studies have revealed that SCRAs with shared structural features, such as indazole rings, are capable of having high affinity, potency, and efficacy at CB1R. For example, Patel et al. ^17^ observed that eleven such SCRAs are highly potent and efficacious unbiased CB1R agonists. This existing work provides evidence that differentiates SCRAs from partial agonists such as THC because indazole-containing SCRAs produce a more stabilizing effect on the “twin toggle switch” that affords CB1R-Gα_i_ interactions as compared to less-stable interactions between the twin toggle switch and THC ^17,20^. It is hoped that the growing body of SCRA literature will afford a better understanding of the structure–activity correlates that underlie toxicities unique to SCRA use as compared to *Cannabis.* Because novel SCRAs with unknown or poorly understood pharmacology continue to be synthetized by illegal markets, gaining additional insight into the pharmacodynamics of these SCRAs is critical to assess the risks of these novel compounds as they enter the marketplace.

The purpose of this study was to assess select emerging SCRAs for their potency, efficacy, binding affinity and differential signaling via CB1R and CB2R. Fifteen SCRAs were assessed in vitro for binding, inhibition of forskolin (FSK)-stimulated cAMP accumulation, and βarrestin2 recruitment at CB1R and CB2R; and in silico to model their unique interactions with CB1R and CB2R. Based on their unique ligand bias, two SCRAs were further assessed in vivo to determine whether observed signaling bias in vitro correlated with specific in vivo drug responses. Our data add to the growing understanding of SCRA structure–activity relationships (SAR) and may extend this SAR into correlations with ligand bias in vitro and in vivo.

## Results

### Type 1 cannabinoid receptor (CB1R)

#### Radioligand binding

**Figure 2:**
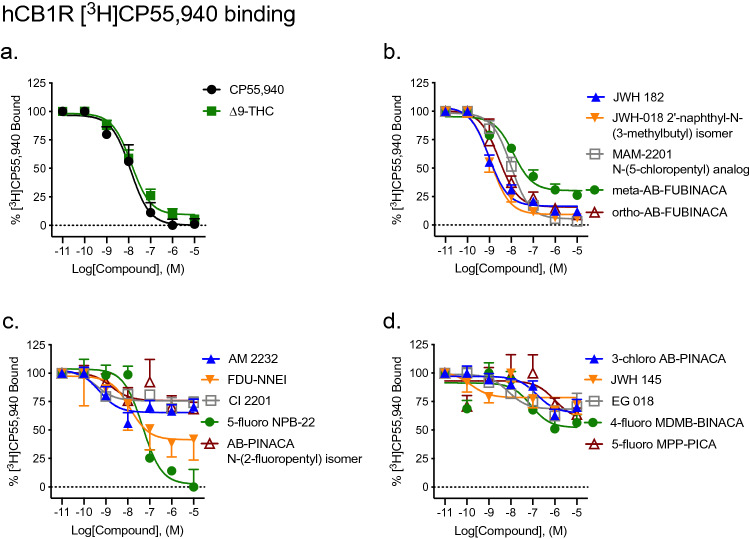
Effect of SCRAs on [^3^H]CP55,940 binding to CB1R. 1 nM [^3^H]CP55,940 to membranes obtained from CHO cells stably-expressing hCB1R. Data were fit to a nonlinear regression (four parameter model, GraphPad v. 8) for statistics in Table 1. Data are mean ± S.E.M. *n* ≥ 6 independent experiments performed in duplicate.

Saturation binding, including non-specific binding performed with CHO-K1 cells not expressing hCB1R, was conducted with [^3^H]CP55,940 to determine *K*_d_ (0.79 [0.24–2.7] nM) and *B*_max_ (1344 ± 32 fmol/mg) (Supplementary Fig. 1a). All fifteen SCRAs displaced 1.0 nM [^3^H]CP55,940 from CB1R. ∆^9^-THC was assayed as a control ligand and displayed similar affinity to that of CP55940 (Fig. 2, Table 1). Ten SCRAs displayed affinity (*K*_i_) that was not different from that of the reference agonist CP55940 at CB1R (Fig. 2, Table 1). Five compounds—JWH 182, JWH 018 2′-naphthyl-N-(3-methylbutyl) isomer, AM 2232, FDU-NNEI, and Cl 2201 displayed significantly greater affinity than that of CP55,940 for CB1R; and one compound—5-fluoro MPP-PICA displayed significantly less affinity than that of CP55,940 for CB1R (Fig. 2, Table 1). Five compounds were able to fully compete 1.0 nM [^3^H]CP55,940 from CB1R: JWH 182, JWH 018 2′-naphthyl-N-(3-methylbutyl) isomer, MAM2201 N-(5-chloropentyl) analog, ortho-AB-FUBINACA, and FDU-NNEI (Fig. 2, Table 1); all other SCRAs only partially displaced [^3^H]CP55,940 (22–77%).

**Table 1:** Activity of SCRAs at CB1R and CB2R.

Compound	CB1R						CB2R					
	[^3^H]CP55,940		cAMP Inhibition		βarrestin2 recruitment		[^3^H]CP55,940		cAMP Inhibition		βarrestin2 recruitment	
	K_i_ (nM)	E_min_ (%)	EC_50_ (nM)	E_max_ (%)	EC_50_ (nM)	E_max_ (%)	K_i_ (nM)	E_min_ (%)	EC_50_ (nM)	E_max_ (%)	EC_50_ (nM)	E_max_ (%)
CP55,940	13 (5.6–33)	0.0 ± 5.2	7.7 (0.13–14)	100 ± 6.2	1500 (960–2500)	100 ± 5.6	29 (13–67)	0.0 ± 5.6	5.0 (0.92–24)	100 ± 9.6	559 (410–761)^†^	100 ± 3.4
∆^9^-THC	14 (7.9–27)	9.1 ± 1.3	36 (21–63)*	72 ± 1.7*	1400 (540–4300)	28 ± 9.8*	31 (15–62)	65 ± 7.6*	13 (2.8–61)	48 ± 3.8*^†^	320 (79–730)	58 ± 1.1*^†^
JWH 182	0.77 (0.45–1.3)*	17 ± 2.2	24 (2.2–280)	78 ± 9.4	1600 (720–3100)	110 ± 9.2	6.2 (2.3–18)	21 ± 1.7*^†^	25 (3.2–210)	120 ± 11^†^	> 10,000^a^	13 ± 1.8*^†^
JWH 018 2'-naphthyl-N-(3-methylbutyl) isomer	0.93 (0.51–1.8)*	9.6 ± 2.7	98 (9.6–310)	80 ± 9.1	> 10,000	4.9 ± 0.51*	7.5 (2.6–41)	54 ± 3.5*^†^	0.52 (0.01–4.2)^†^	110 ± 9.2	> 10,000	6.1 ± 1.3*
MAM2201 N-(5-chloropentyl) analog	10 (5.9–17)	5.3 ± 3.0	2.8 (0.83–9.4)	98 ± 8.7	> 10,000	17 ± 2.7*	> 10,000^a^	40 ± 3.8*^†^	580 (41–1400)*^†^	120 ± 25	> 10,000	5.5 ± 1.9*
meta-AB-FUBINACA	16 (7.1–37)	30 ± 2.9*	5.7 (0.64–100)	91 ± 12	> 10,000	22 ± 6.9*	0.63 (0.15–7.4)*	89 ± 6.3*^†^	5.4 (2.2–14)	86 ± 13	280 (93–430)^a^	130 ± 3.7*^†^
ortho-AB-FUBINACA	2.7 (0.77–10)	16 ± 5.4	3.6 (0.35–31)	83 ± 8.4	810 (560–1100)	280 ± 11*	0.93 (0.04–2.4)*	17 ± 1.2	0.06 (0.001–3.1)	110 ± 6.7	140 (44–460)^†^	120 ± 3.1*^†^
AM 2232	0.31 (0.001–2.9)*	66 ± 3.6*	190 (67–700)*	100 ± 9.3	> 10,000	68 ± 1.6*	600 (430–1100)*	73 ± 6.2*	1.7 (0.48–16)^†^	86 ± 1.9	130 (45–370)*	130 ± 19*^†^
FDU-NNEI	14 (2.2–71)	41 ± 2.8	1.0 (0.12–9.8)	96 ± 8.1	> 10,000	49 ± 8.2*	8.8 (1.8–39)	85 ± 8.1*^†^	0.01 (0.001–7.1)	103 ± 7.1	1000 (390–1300)	25 ± 1.3*^†^
Cl 2201	0.09 (0.001–5.4)*	76 ± 2.8*	2.6 (1.2–5.8)	89 ± 3.9	> 10,000	16 ± 1.0*	26 (7.8–82)	66 ± 2.5*	1.0 (0.07–8.3)	110 ± 8.4	120 (33–560)	17 ± 1.1*
5-fluoro NPB-22	47 (20–110)	2.3 ± 7.8	53 (8.2–200)	96 ± 7.0	830 (500–1300)	210 ± 12*	4.3 (0.78–24)	34 ± 8.1*^†^	3.2 (1.3–7.5)	110 ± 7.6	280 (65–580)	120 ± 3.7*^†^
AB-PINACA N-(2-fluoropentyl) isomer	1.6 (0.08–11)	76 ± 6.2*	7.3 (0.61–100)	75 ± 7.5*	240 (140–450)*	204 ± 10*	270 (97–780)*	2.8 ± 8.1^†^	10 (1.8–46)	91 ± 3.2	9.4 (3.2–27)*^†^	140 ± 12*^†^
3-chloro AB-PINACA	170 (24–740)	65 ± 5.1*	0.04 (0.001–0.22)	94 ± 2.8	140 (93–200)*	340 ± 11*	52 (8.9–94)	84 ± 4.3*	19 (5.9–110)^†^	87 ± 15	8.8 (3.0–24)*^†^	130 ± 4.5*^†^
JWH 145	110 (21–800)	69 ± 7.6*	0.03 (0.001–1.5)	82 ± 9.3	> 10,000	27 ± 9.0*	860 (560–1300)*	70 ± 6.1*	61 (2.7–200)^†^	86 ± 17	190 (37–520)	15 ± 1.7*
EG 018	8.6 (0.68–76)	68 ± 6.1*	450 (69–2900)*	104 ± 18	> 10,000	5.2 ± 0.54*	1.9 (0.27–8.0)*	53 ± 4.0*	51 (5.2–580)	85 ± 17	770 (350–1700)	10 ± 1.9*
4-fluoro MDMB-BINACA	99 (14–890)	52 ± 7.5*	10 (1.8–83)	87 ± 5.6	88 (49–150)*	240 ± 10*	0.41 (0.02–8.1)*	57 ± 11*	0.56 (0.05–22)	95 ± 11	120 (53–250)*	130 ± 8.2*^†^
5-fluoro MPP-PICA	1200 (160–3200)*	59 ± 18*	6.4 (1.3–30)	92 ± 6.8	> 10,000	170 ± 9.8*	540 (150–780)*	89 ± 6.3*	12 (1.5–77)	110 ± 13	630 (250–1100)^a^	82 ± 1.9*^†^

**Figure 3:**
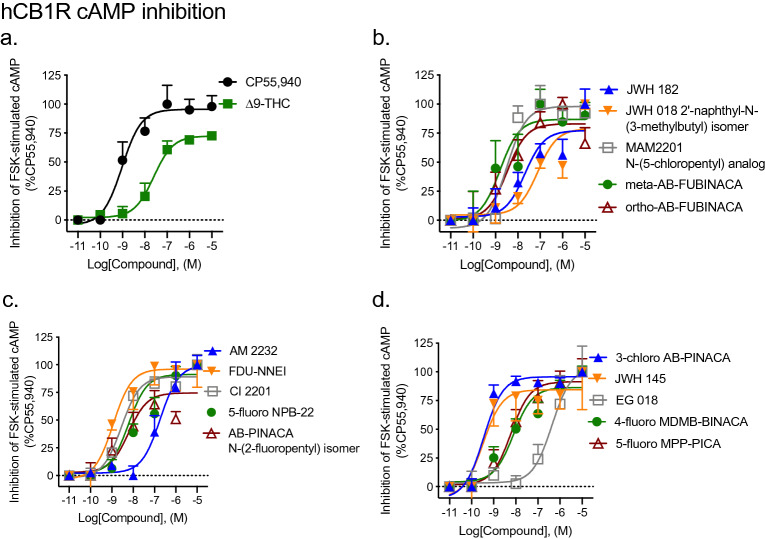
CB1R-dependent inhibition of FSK-stimulated cAMP following SCRA treatment. CHO cells stably-expressing hCB1R were treated with 10 µM FSK and 0.10 nM–10 μM SCRA for 90 min and inhibition of cAMP was measured. cAMP inhibition data are expressed as % CP55,940 response. Data were fit to a nonlinear regression (four parameter model, GraphPad v. 8) for statistics in Table 1; or fit to the operational model to calculate bias (Fig. 5). Data are mean ± S.E.M. *n* ≥ 6 independent experiments performed in triplicate.

**Figure 4:**
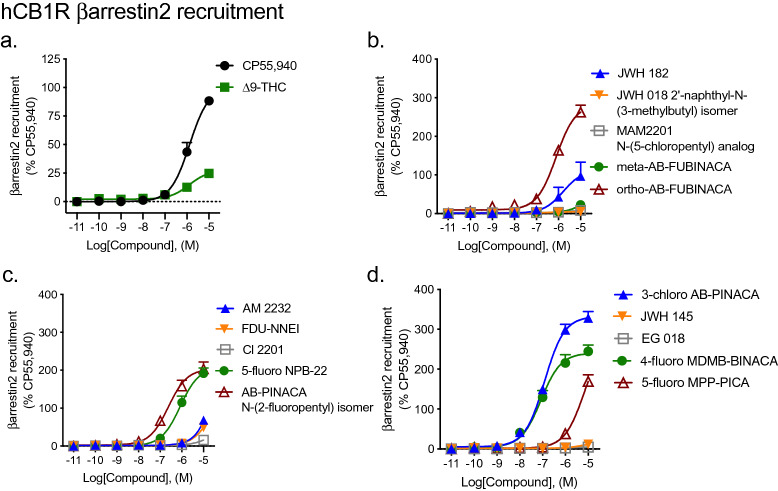
βarrestin2 recruitment to CB1R following SCRA treatment. CHO cells stably-expressing hCB1R were treated with 0.10 nM–10 μM SCRA 90 min and βarrestin2 recruitment was measured. βarrestin2 recruitment data are expressed as % CP55,940 response. Data were fit to a nonlinear regression (four parameter model, GraphPad v. 8) for statistics in Table 1; or fit to the operational model to calculate bias (Fig. 5). Data are mean ± S.E.M. *n* ≥ 6 independent experiments performed in triplicate. Note the difference in y-axis scale between (**a**) and all other panels.

Compound activity was quantified for [^3^H]CP55,940 binding, inhibition of FSK-stimulated cAMP, or βarrestin2 recruitment in CHO cells stably expressing CB1R or CB2R and treated with SCRAs. Data were fit to a variable slope (four parameter) non-linear regression in GraphPad (v. 8). n ≥ 6 independent experiments performed in triplicate from Figs. 2, 3, 4, 7, 8, and 9. *E*_Max_ and *E*_Min_ refer to the top and bottom of the concentration–response curves, respectively. Data are expressed as nM with 95% CI or %CP55,940 response, mean ± SEM.

*p < 0.05 compared to CP55,940 within assay and measurement as determined via non-overlapping 95% CI or one-way ANOVA followed by Tukey's post-hoc test.

^†^p < 0.05 compared to CB1R response within compound and measurement as determined via non-overlapping 95% CI or one-way ANOVA followed by Tukey's post-hoc test.

^a^Indicates a compound displayed activity for one receptor but not the other (no statistical comparison).

#### Inhibition of FSK-stimulated cAMP

All fifteen SCRAs displayed agonist activity for the inhibition of FSK-stimulated cAMP at CB1R. The majority of these were full agonists of CB1R-dependent cAMP inhibition relative to the reference agonist CP55940, and displayed similar potency compared to that of CP55940 as well (Fig. 3, Table 1). ∆^9^-THC was assayed as a control partial agonist and displayed significantly lower efficacy and potency relative to CP55940 (Fig. 3, Table 1). One SCRA—AB-PINACA N-(2-fluoropentyl) isomer—was a partial agonist of CB1R-dependent cAMP inhibition relative to CP55940, but retained high potency (Fig. 3, Table 1). Two compounds—AM 2232 and EG 018—displayed significantly lower potency relative to CP55940, but retained full (i.e., 100%) efficacy (Fig. 3, Table 1). Importantly, cAMP accumulation was not completely inhibited by the maximum effects observed with SCRAs, indicating that supramaximal responses could have been detected had they occurred (Supplementary Fig. 2a).

#### βarrestin2 recruitment

Nine of fifteen SCRAs displayed remarkably weak (i.e. low efficacy and potency > 10,000 nM) agonist activity for the recruitment of βarrestin2 to CB1R relative to CP55940 (Fig. 4, Table 1). ∆^9^-THC was assayed as a control partial agonist and displayed significantly lower efficacy, but similar potency, relative to CP55940 (Fig. 4, Table 1). JWH 182, ortho-AB-FUBINACA, and 5-fluoro NPB-22 were equipotent, but produced supramaximal *E*_Max_ responses (i.e. > 100%) relative to CP55940 (Fig. 4, Table 1). AB-PINACA N-(2-fluoropentyl) isomer, 3-chloro AB-PINACA, and 4-fluoro MPP-PICA displayed greater potency and produced supramaximal *E*_Max_ responses (i.e. > 100%) relative to CP55940 (Fig. 4, Table 1).

#### Bias analyses

**Figure 5:**
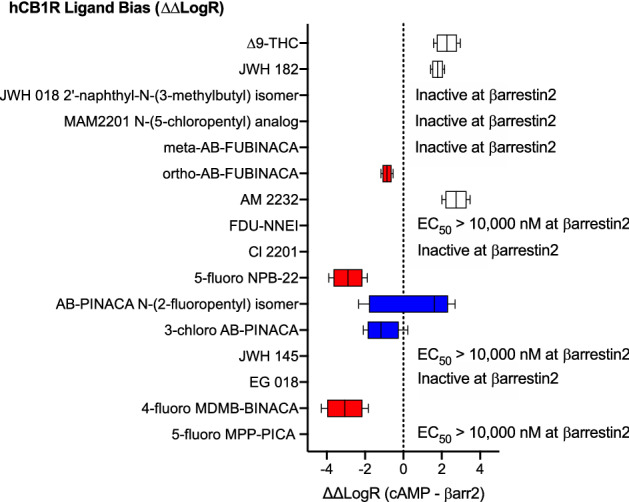
CB1R SCRA bias. Compound bias between cAMP inhibition and βarrestin2 recruitment [ΔΔLogR (cAMP—βarr2)] is shown here for data presented in Figs. 3 and 4; Table 1. Data were fit to the operational model to calculate bias. Data are mean with 95% CI, *n* ≥ 6 independent experiments performed in triplicate. Values > 0 represent cAMP bias (white), values < 0 represent βarrestin2 bias (red), and values not different from 0 are unbiased (blue) as determined by 95% CI not overlapping with 0.

Given the high potency and efficacy observed for most SCRAs in the cAMP inhibition assay and comparatively low potency and efficacy observed for the majority of these compounds in the βarrestin2 recruitment assay. It is not surprising that most of the SCRAs tested displayed bias toward the inhibition of cAMP relative to βarrestin2 recruitment (i.e. ∆∆LogR > 0) (Fig. 5). Two SCRAs—AB-PINACA N-(2-fluoropentyl) isomer, 3-chloro AB-PINACA did not display a bias for cAMP inhibition versus βarrestin2 recruitment (i.e. ∆∆LogR not different from 0) (Fig. 5). Finally, three compounds—ortho-AB-FUBINACA, 4-fluoro MDMB-BINACA, and 5-fluoro NPB-22 displayed a significant bias toward the recruitment of βarrestin2 relative to inhibition of FSK-stimulated cAMP (i.e. ∆∆LogR < 0) (Fig. 5).

### Type 2 cannabinoid receptor (CB2R)

#### Radioligand binding

**Figure 6:**
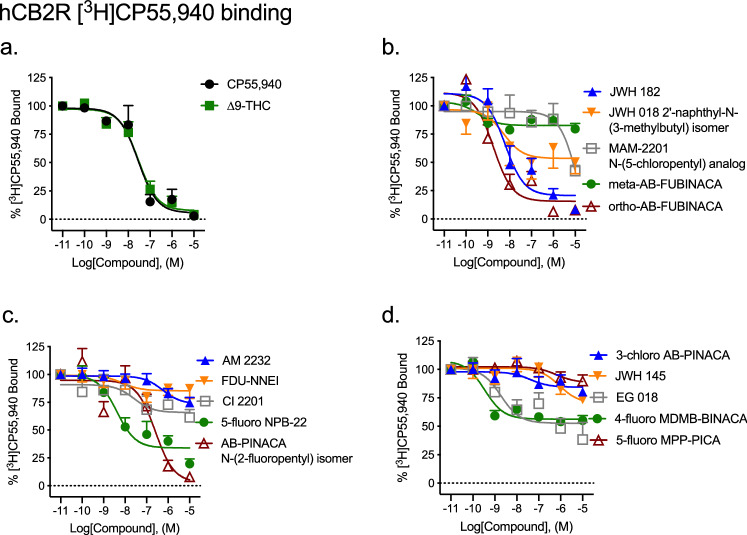
Effect of SCRAs on [^3^H]CP55,940 binding to CB2R. 1 nM [^3^H]CP55,940 to membranes obtained from CHO cells stably-expressing hCB2R. Data were fit to a nonlinear regression (four parameter model, GraphPad v. 8) for statistics in Table 1. Data are mean ± S.E.M. *n* ≥ 6 independent experiments performed in duplicate.

Saturation binding in CHO-K1 cells stably expressing hCB2R, as well as non-specific binding performed with CHO-K1 cells not expressing hCB2R, was conducted with [3H]CP55,940 to determine *K*_d_ (0.93 [0.29–1.9] nM) and *B*_max_ (1447 ± 50 fmol/mg) (Supplementary Fig. 1b). All fifteen SCRAs displaced 1.0 nM [3H]CP55,940 from CB2R. ∆^9^-THC was assayed as a control ligand and did fully compete [3H]CP55,940 from the receptor (Fig. 6, Table 1). Six SCRAs displayed affinity (*K*_i_) that was not different from that of the reference agonist CP55,940 at CB2R (Fig. 6, Table 1). Four compounds (meta-AB-FUBINACA, ortho-AB-FUBINACA, EG 018, and 4-fluoro MDMB-BINACA) displayed significantly greater affinity than that of CP55,940 for CB2R; and five compounds (MAM2201 N-(5-chloropentyl) analog, AM 2232, AB-PINACA N-(2-fluoropentyl) isomer, JWH 145, and 5-fluoro MPP-PICA) displayed significantly less affinity than that of CP55,940 for CB2R (Fig. 6, Table 1). Of these, MAM2201 N-(5-chloropentyl) analog displayed very low affinity (> 10,000 nM). Only two SCRAs completely displaced 1.0 nM [^3^H]CP55,940 from CB2R: ortho-AB-FUBINACA and AB-PINACA N-(2-fluoropentyl) isomer (Fig. 6, Table 1); all other SCRAs only partially displaced [^3^H]CP55,940 (21–89%).

#### Inhibition of FSK-stimulated cAMP

**Figure 7:**
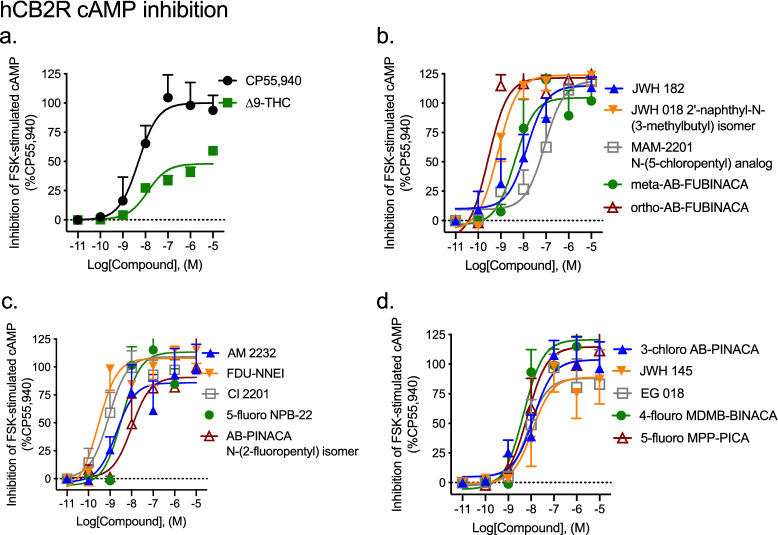
CB2R-dependent inhibition of FSK-stimulated cAMP following SCRA treatment. CHO cells stably-expressing hCB2R were treated with 10 µM FSK and 0.10 nM–10 μM SCRA for 90 min, and inhibition of cAMP was measured. cAMP inhibition data are expressed as % CP55,940 response. Data were fit to a nonlinear regression (four parameter model, GraphPad v. 8) for statistics in Table 1; or fit to the operational model to calculate bias (Fig. 9). Data are mean ± S.E.M. *n* ≥ 6 independent experiments performed in triplicate.

All fifteen SCRAs displayed full agonist activity to inhibit FSK-stimulated cAMP at CB2R (i.e. not different from 100%) (Fig. 7, Table 1). With one exception, MAM2201 N-(5-chloropentyl) analog, the potency of these compounds at CB2R for cAMP inhibition was not different compared to that of CP55,940 (Fig. 7, Table 1). ∆^9^-THC was assayed as a control partial agonist and displayed significantly lower efficacy relative to CP55,940 (Fig. 7, Table 1). As was observed for CB1R, cAMP accumulation was not completely inhibited by the maximum effects observed with SCRAs, indicating that supramaximal responses could have been detected had they occurred (Supplementary Fig. 2b).

#### βarrestin2 recruitment

**Figure 8:**
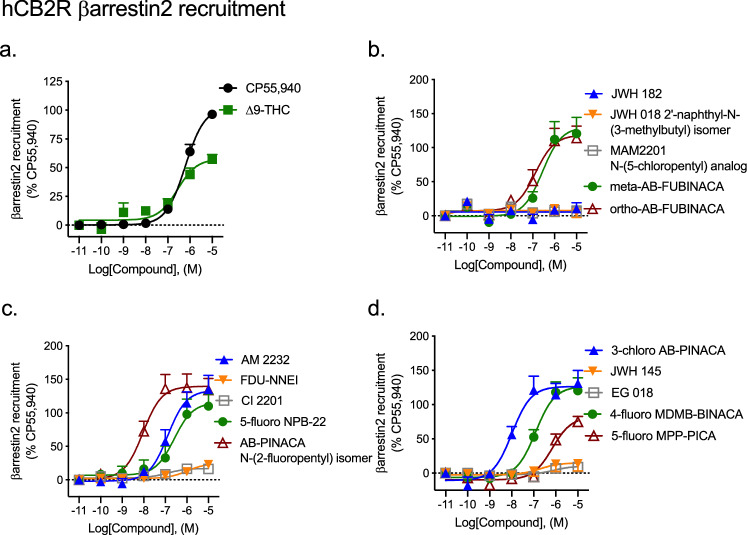
βarrestin2 recruitment to CB2R following SCRA treatment. CHO cells stably-expressing hCB2R were treated with 0.10 nM–10 μM SCRA 90 min and βarrestin2 recruitment was measured. βarrestin2 recruitment data are expressed as % CP55,940 response. Data were fit to a nonlinear regression (four parameter model, GraphPad v. 8) for statistics in Table 1; or fit to the operational model to calculate bias (Fig. 9). Data are mean ± S.E.M. *n* ≥ 6 independent experiments performed in triplicate. Note the difference in y-axis scale between a and all other panels.

Seven of fifteen SCRAs displayed none or very weak (i.e. low efficacy and EC_50_ > 10,000 nM) agonist activity for the recruitment of βarrestin2 to CB2R relative to CP55,940 (Fig. 8, Table 1). ∆^9^-THC was assayed as a control partial agonist and displayed significantly lower efficacy, but similar potency, relative to CP55,940 (Fig. 8, Table 1). Four compounds: meta-AB-FUBINACA, ortho-AB-FUBINACA, 5-fluoro NPB-22, and 5-fluoro MPP-PICA were not different with regard to potency relative to the reference agonist CP55,940, and produced supramaximal *E*_Max_ responses (i.e. > 100%) relative to CP55,940 (Fig. 8, Table 1). In contrast, five other compounds AM 2232, AB-PINACA N-(2-fluoropentyl) isomer, 3-chloro AB-PINACA, and 4-fluoro MDMB-BINACA displayed greater potency and produced supramaximal *E*_Max_ responses (i.e. > 100%) relative to CP55,940 (Fig. 8, Table 1).

#### Bias analyses

**Figure 9:**
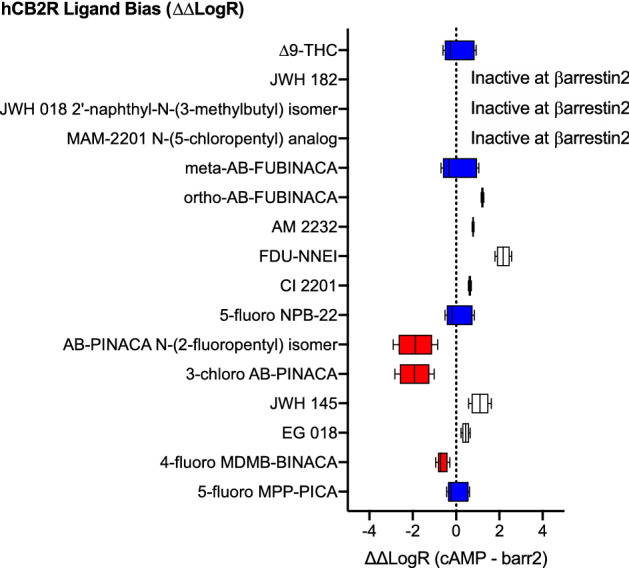
CB2R SCRA bias. Compound bias between cAMP inhibition and βarrestin2 recruitment (ΔΔLogR (cAMP—βarr2)] is shown here for data presented in Figs. 7 and 8; Table 1. Data were fit to the operational model to calculate bias. Data are mean with 95% CI, *n* ≥ 6 independent experiments performed in triplicate. Values > 0 represent cAMP bias (white), values < 0 represent βarrestin2 bias (red), and values not different from 0 are unbiased (blue) as determined by 95% CI not overlapping with 0.

As with CB1R signaling, SCRAs were assessed for bias toward the inhibition of cAMP relative to βarrestin2 recruitment (i.e. ∆∆LogR > 0) at CB2R (Fig. 9). THC and three SCRAs—meta-AB-FUBINACA, 5-fluoro NPB-22, and 5-fluoro MPP-PICA did not display a bias for cAMP inhibition versus βarrestin2 recruitment (i.e. ∆∆LogR not different from 0) (Fig. 9). Three compounds were inactive in βarrestin2 and were therefore considered obligate cAMP-biased ligands: JWH 182, JWH 018 2′-napthyl-N-(3-methylbutyl) isomer, and MAM2201 N-(5-chloropentyl) analog (Fig. 9). Six compounds—ortho-AB-FUBINACA, AM 2232, FDU-NNEI, Cl 2201, JWH 145, and EG 018 displayed a significant bias toward the inhibition of cAMP versus recruitment of βarrestin2 (i.e. ∆∆LogR > 0) (Fig. 9) Finally, three compounds—AB-PINACA N-(2-fluoropentyl isomer, 3-chloro AB-PINACA, and 4-fluoro MDMB-BINACA—displayed a significant bias toward the recruitment of βarrestin2 relative to inhibition of FSK-stimulated cAMP (i.e. ∆∆LogR < 0) (Fig. 5).

### CB1R versus CB2R selectivity

With regard to receptor affinity, six SCRAs displayed greater affinity at CB1R relative to CB2R: JWH 018 2′-naphthyl-N-(3-methylbutyl) isomer, MAM2201 N-(5-chloropentyl) analog, AM 2232, FDU-NNEI, Cl 2201, and AB PINACA N-(2-fluoropentyl) isomer (Figs. 1, 6; Table 1). Interestingly, AB PINACA N-(2-fluoropentyl) isomer was unique in displaying a higher affinity for CB1R but only partially competing for [^3^H]CP55,940 binding at CB1R, whereas this SCRA fully competed with [^3^H]CP55,940 at CB2R (Figs. 1, 6; Table 1). Only one SCRA—4-fluoro MDMB-BINACA—displayed greater affinity at CB2R relative to CB1R (Figs. 1, 6; Table 1). FDU-NNEI displaced more [^3^H]CP55,940 at CB1R relative to CB2R (Figs. 1, 6; Table 1).

In the cAMP inhibition assay, several compounds displayed receptor subtype selectivity with regard to potency and efficacy. JWH 018 2′-naphthyl-N-(3-methylbutyl) isomer and AM 2232 were more potent at CB2R relative to CB1R; whereas MAM2201 N-(5-chloropnetyl) analog, 3-chloro AB-PINACA, and JWH 145 were more potent at CB1R relative to CB2R. JWH 182 displayed greater efficacy at CB2R than CB1R (Table 1).

In the βarrestin2 recruitment assay, meta-AB FUBINACA and 5-fluoro MPP-PICA were minimally active at CB1R but agonists at CB2R. Conversely, JWH 182 was inactive at CB2R but an agonist at CB1R (Table 1). Ortho-AB-FUBINACA, AB-PINACA N-(2-fluoropentyl) isomer, and 3-chloro AB-PINACA were more potent for CB2R-dependent recruitment of βarrestin2 than CB1R-dependent recruitment of βarrestin2 (Table 1). Eight SCRAs displayed significantly less efficacy for CB2R-dependent βarrestin2 recruitment relative to CB1R-dependent βarrestin2 recruitment, which is likely attributable to the supramaximal responses observed for several SCs at CB1R in this assay (Table 1).

### In silico docking to CB1R and CB2R

**Figure 10:**
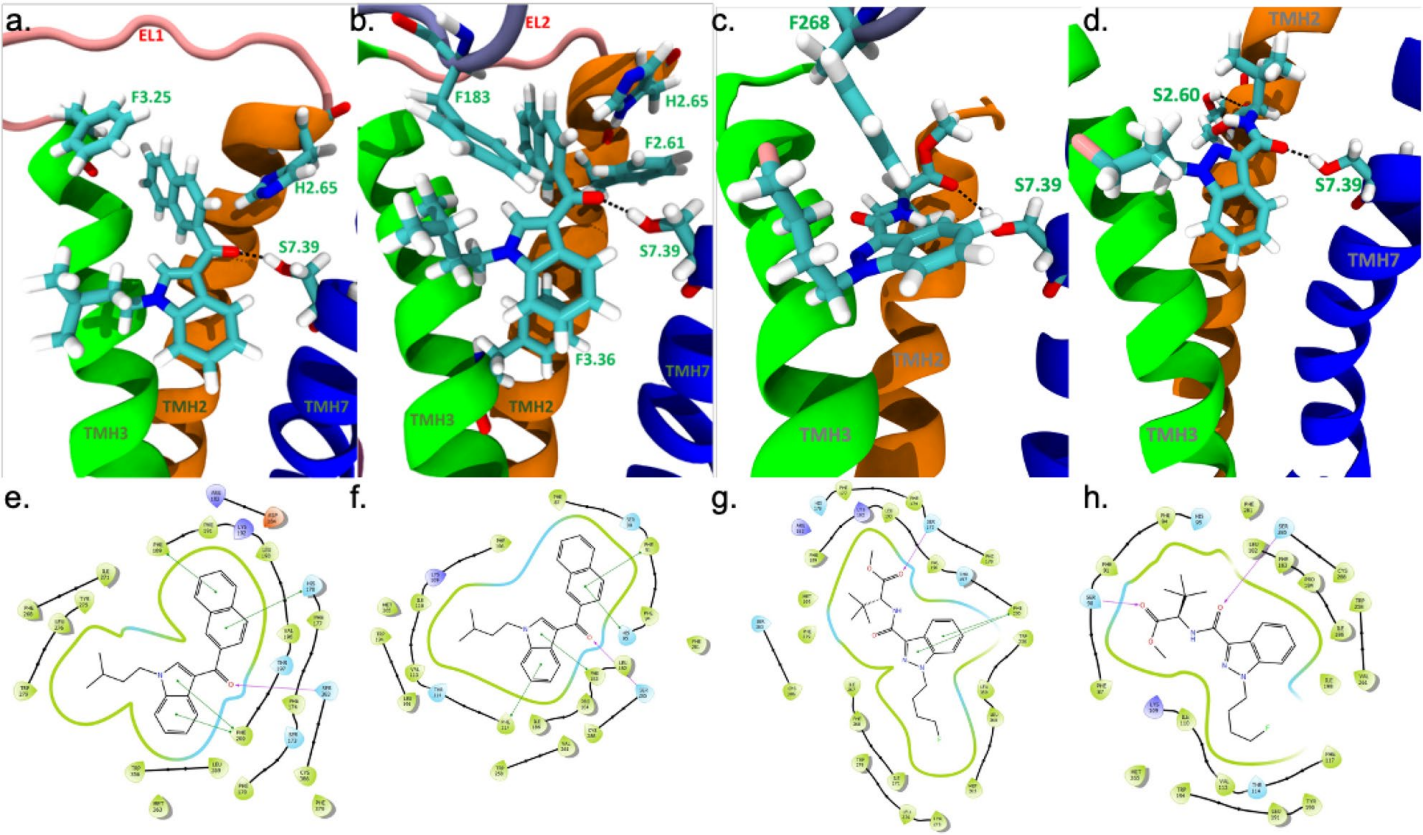
Representative models of SCRAs bound to CB1R and CB2R. 3D renderings of JWH 018 2′-naphthyl-N-(3-methylbutyl) isomer bound to CB1R PDB: 6N4B (**a**) and CB2R PBD: 6PT0 (**b**). 3D renderings of 4-fluoro MDMB-BINACA bound to CB1R PDB: 6N4B (**c**) and CB2R PBD: 6PT0 (**d**). Ligands and interacting amino acid residues are shown in cyan. Amino acid interacting partners are labelled in green and with Ballesteros and Weinstein numbering. Hydrogen bonds are shown by black dotted lines. Planar renderings of JWH 018 2′-naphthyl-N-(3-methylbutyl) isomer bound to CB1R PDB: 6N4B (**e**) and CB2R PBD: 6PT0 (**f**). 3D renderings of 4-fluoro MDMB-BINACA bound to CB1R PDB: 6N4B (**g**) and CB2R PBD: 6PT0 (**h**). Interacting amino acid residues that form the orthosteric binding pocket are shown in green (hydrophobic interactions) or blue (polar interactions). Green lines indicate π–π interacting partners, maroon arrows indicate hydrogen bonds, and black lines connecting amino acids indicate shared transmembrane helices. All images were produced through in silico docking in Schrödinger program by the authors.

Following our cell culture-based work, we wanted to determine whether the structures of these SCRAs might explain their ligand bias and receptor subtype selectivity. Binding of each SCRA to CB1R and CB2R was modelled via induced fit docking in Schrödinger using G protein-bound structures for each receptor (CB1R PDB 6N4B ^20^; CB2R PBD 6PT0 ^21^). All fifteen cannabinoids studied bound to the orthosteric sites of CB1R and CB2R using these models (JWH 018 2′-naphthyl-N-(3-methylbutyl) isomer and 4-fluoro MDMB-BINACA shown in Fig. 10). Two interactions were conserved between both CB1R and CB2R in terms of the binding of SCRAs: the hydrogen bond with Ser383^7.39^ (CB1R) or Ser285^7.39^ (CB2R) and the T-shaped π–π interaction His178^2.65^ (CB1R) and His95^2.65^ (CB2R). Furthermore, many common interactions were observed between homologs amino acid residues at CB1R and CB2R, which suggests that the binding sites are well conserved between both receptors (Tables 2 and 3).

**Table 2:** Summary of the interactions between the residues of the CB1R (PDB: 6N4B) and each of the SCRAs.

Compound	F170^2.57^ π–π	S173^2.60^ H-bond	F177^2.64^ π–π	H178^2.65^ H-bond	H178^2.65^ π–π	R182^EC1^ Halogen	F189^3.25^ π–π	F200^3.36^ π–π	F268^EL2^ π–π	W279^5.43^ π–π	L376^7.32^ H-bond	S383^7.39^ H-bond	Interaction energy (kcal/mol)
JWH-182					X			X	X	X		X	− 132.75
JWH 018 2′-naphthyl-N-(3-methylbutyl) isomer					X		X					X	− 118.41
MAM2201 N-(5-chloropentyl) analog			X		X			X	X			X	− 128.37
meta AB-FUBINACA										X		X	− 121.84
ortho AB-FUBINACA				X				X	X	X		X	− 117.77
AM 2232	X								X		X		− 113.89
FDU-NNEI			X	X			X		X	X		X	− 114.64
Cl 2201			X		X	X		X	X			X	− 123.69
5-fluoro NPB-22			X				X	X	X	X		X	− 112.59
AB-PINACA N-(2-fluoropentyl) isomer		X	X										− 116.02
3-Chloro AB-PINACA		X						X					− 112.12
JWH 145									X	X			− 134.03
EG 018		X	X				X		X				− 126.92
4-Fluoro MDMB-BINACA	X							X					− 105.63
5-Fluoro MPP-PICA		X						X				X	− 107.13

The right column lists the interaction energy of each SCRA.

**Table 3:** Summary of the interactions between the residues of the CB2R (PDB: 6PT0) and each SCRA.

Compound	F87^2.57^ H-bond	S90^2.60^ H-bond	F91^2.61^ π–π	F94^2.64^ π–π	H95^2.65^ π–π	T114^3.33^ π–π	F117^3.36^ π–π	F183^EL2^ π–π	W194^5.43^ π–π	W258^6.48^ π–π	S285^7.39^ H-bond	Interaction energy (kcal/mol)
JWH-182						X	X	X	X		X	− 119.80
JWH 018 2′-naphthyl-N-(3-methylbutyl) isomer			X		X		X	X			X	− 111.40
MAM2201 N-(5-chloropentyl) analog			X		X			X			X	− 126.21
meta AB-FUBINACA	X	X									X	− 124.22
ortho AB-FUBINACA	X	X							X		X	− 118.99
AM 2232			X								X	− 114.25
FDU-NNE1								X			X	− 116.92
Cl 2201		X	X		X						X	− 122.02
5-Fluoro NPB-22			X	X						X	X	− 123.90
AB-PINACA N-(2-fluoropentyl) isomer		X									X	− 105.84
3-Chloro AB-PINACA	X	X					X				X	− 115.84
JWH 145	X				X				X		X	− 128.12
EG 018			X		X						X	− 129.34
4-Fluoro MDMB-BINACA								X			X	− 112.01
5-Fluoro MPP-PICA		X		X				X		X	X	− 119.45

The right column lists the interaction energy of each SCRA.

Because the amino acid sequence and binding sites are well conserved between CB1R and CB2R, the interaction energy calculated for each cannabinoid represents a predictive metric that can be used to estimate and compare the relative affinity of each cannabinoid at each receptor (Tables 2 and 3). This metric would predict that JWH-182, JWH 018 2′-naphthyl-N-(3-methylbutyl) isomer, MAM2201 N-(5-chloropentyl) analog, Cl 2201, AB-PINACA N-(2-fluoropentyl) isomer, JWH 145, and 4-fluoro MDMB-BINACA would have greater affinity at CB1R. While meta-AB-FUBINACA, ortho-AB-FUBINACA, AM 2232, FDU-NNEI, 5-fluoro NPB-22, 3-chloro AB-PINACA, EG 018 and 5-fluoro MPP-PICA would have greater affinity at CB2R. These modelled predictions are largely in agreement with our experimental findings in CHO-K1 cells, indicating good validity of the modelled receptors. However, three notable exceptions were found in our data: (1) 4-fluoro MDMB-BINACA displayed a higher affinity for CB2R in [^3^H]CP55,940 binding assays, but greater affinity was predicted to CB1R in this model, and greater potency and efficacy was observed for this compound at CB1R for βarrestin2 recruitment; (2) AM 2232 displayed a higher affinity for CB1R in [^3^H]CP55,940 binding assays, but cAMP inhibition and modeling data suggest this compound is CB2R-selective; and (3) FDU-NNEI displayed a higher affinity for CB1R in [^3^H]CP55,940 binding assays, but greater affinity was predicted for CB2R in these models.

Using these models and focusing on CB1R, if we compare the highest affinity CB1R agonist (Cl 2201) to the weakest affinity CB1R agonist (JWH 145) we can understand the structural features that make a high affinity CB1R agonist. Firstly, the main structural difference between Cl 2201 and JWH 145 is that in Cl 2201 the benzene ring is fused to the pyridine vs in JWH 145 it is a branched benzene ring. Because of this, the two cannabinoids only share one similar interaction: Phe268^EL2^. In fact, JWH 145 only has two electrostatic interactions with CB1R. However, if we compare Cl 2201 with MAM2201 N-(5-chloropentyl) analog, which are very similar in structure, we can likely see what interactions are important for Cl 2201’s high affinity for CB1R. The major difference between these two structures is that MAM 2201 N-(5-chloropentyl) analog has a methyl substitution from the naphthalene group, whereas Cl 2201 has a chlorine substitution at this position. MAM 2201 also bears a choro-pentyl whereas Cl 2201 has a fluorine. The binding poses of these cannabinoids are identical except for the Arg182^EC1^ halogen bond exhibited by Cl 2201 but not by MAM2201. Thus, this halogen bond with extracellular loop one may give rise to the high affinity of Cl 2201 at CB1R relative to other SCRAs.

We also attempted to model ligand affinity to CB1R in either G protein-permissive or G protein-absent states using these in silico models and two selective highly biased CB1R SCRAs: JWH 018 2′-naphthyl-N-(3-methylbutyl) isomer (lacking βarrestin2 activity) (Fig. 10a,e) and 4-fluoro MDMB-BINACA (βarrestin2 biased) (Fig. 10c,g). Modelling was carried out with CB1R 6N4B in the presence and absence of Gα_i_ in order to simulate a G protein-accommodating state (i.e. a model of G protein bias) and a non-G protein-accommodating state (i.e. a model of non-G protein bias). JWH 018 2′-naphthyl-N-(3-methylbutyl) isomer bound with greater estimated affinity to CB1R with Gα_i_ present (greater negative interaction energy); this is congruent with the ligand’s G protein bias (Table 4). 4-fluoro MDMB-BINACA, on the other hand, bound with greater estimated affinity to CB1R without Gα_i_ present; this is congruent with the ligand’s observed βarrestin2 bias (Table 4). These in silico assessments of SCRA affinity with and without G protein serve as validation of our cell culture experiments, but do not themselves represent a determination of bias. Overall, in silico modeling of SCRAs to cannabinoid receptors was largely congruent with cell signaling experiments but cannot fully model the dynamic and multi-faceted aspects of ligand-receptor interaction necessary to predict receptor subtype selectivity.

**Table 4:** Modelling of JWH 018 2′-naphthyl-N-(3-methylbutyl) isomer and 4-fluoro MDMB-BINACA bound to 6N4B with and without G protein.

Compound	Interaction energy (kcal/mol), G protein removed	Interaction energy (kcal/mol), G protein included	Observed CB1R bias in cell culture
JWH 018 2′-naphthyl-N-(3-methylbutyl) isomer	− 114.63	− 118.41	G protein
4-Fluoro MDMB-BINACA	− 116.19	− 105.63	βarrestin2

### In vivo activity

**Figure 11:**
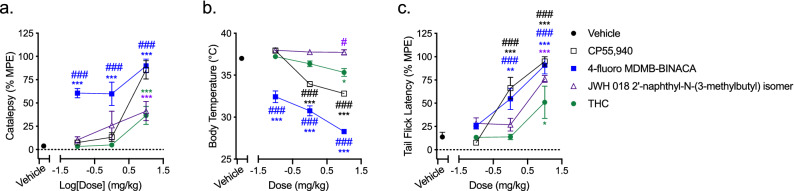
SCRA modulation of catalepsy, body temperature and nociception in vivo. Male C57Bl/6 mice were treated with 0.1–10 mg/kg *i.p.* SCRA or vehicle (1:1:18 ethanol:emulphor:saline). Physiological assessments of (**a**) catalepsy (5 min, % MPE 60 s), (**b**) body temperature (15 min), and (**c**) nociception in the tail flick assay (52 °C) (20 min, % MPE 20 s) were then made. Data are mean ± S.E.M, *n* = 6 animals/treatment. *p < 0.05, **p < 0.01, ***p < 0.001 relative to vehicle; ^#^*p* < 0.05, ^###^*p* < 0.001 relative to THC within dose according to the matched colour for each dataset (two-way ANOVA followed by Bonferroni’s post-hoc test).

Finally, we wanted to determine whether CB1R SCRAs with observable βarrestin2 bias in cell culture and in silico experiments produced an in vivo effect profile that was distinct from G protein-biased and non-biased ligands. To do so, two compounds were assessed in a triad of cannabinoid-based outcomes in adult, male, C57Bl/6 mice: the highly βarrestin2-biased 4-fluoro MDMB-BINACA and JWH 018 2′-napthyl-N-(3-methylbutyl) isomer, which was inactive in the CB1R βarrestin2 assay. CP55,940 and THC were also assessed as positive controls for full and partial agonism at CB1R in vivo, respectively (Fig. 11). Time points for in vivo activity of *i.p.* administered cannabinoids were chosen to be 5 min, 15 min, and 20 min for catalepsy, body temperature, and nociception, respectively, based on the previously-published findings of ourselves and others ^19,22,23^ and time course experiments conducted with CP55,940 (Supplementary Fig. 3). 4-fluoro MDMB-BINACA was a full agonist of all three measured responses: catalepsy (Fig. 11a), hypothermia (Fig. 11b), and anti-nociceptive response (Fig. 11c). Producing substantial increases in catalepsy and reductions in body temperature at even the lowest doses tested (0.1 mg/kg *i.p.*) and producing effects in all three responses that were greater than THC. The responses to 4-fluoro MDMB-BINACA were greater than those observed for CP55,940 in catalepsy and body temperature measurements, but not different in the anti-nociception assay (Fig. 11). In contrast to 4-fluoro MDMB-BINACA, JWH 018 2′-napthyl-N-(3-methylbutyl) isomer was a partial agonist in the catalepsy assay (Fig. 11a), did not change body temperature (Fig. 11b), and was a full agonist in the anti-nociception assay (Fig. 11c).

**Figure 12:**
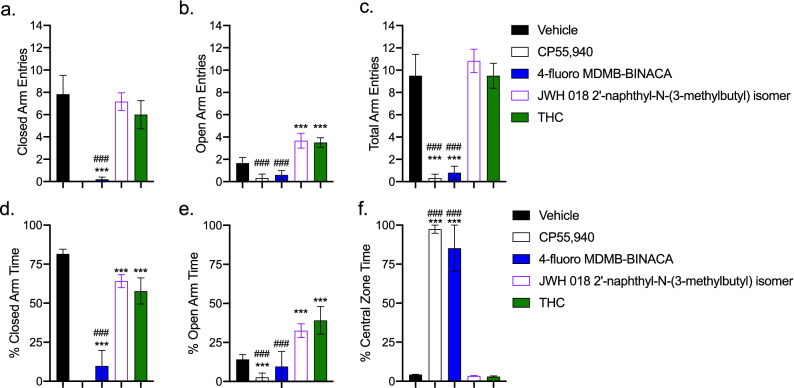
SCRA activity in the elevated plus maze (EPM). Male C57Bl/6 mice were treated with 1.0 mg/kg *i.p.* SCRA or vehicle (1:1:18 ethanol:emulphor:saline). EPM was conducted 30 min after ligand treatment for 5 min. Mice were assessed for closed (**a**), open (**b**), and total arm entries (**c**); as well as the % time spent in the closed (**d**) and open arms (**e**) and the central zone of the EPM (**f**). Data are mean ± S.E.M, *n* = 5–6 animals/treatment. ***p < 0.001 relative to vehicle; ^###^*p* < 0.001 relative to THC for each dataset (two-way ANOVA followed by Bonferroni’s post-hoc test).

The SCRAs 4-fluoro MDMB-BINACA and JWH 018 2′-napthyl-N-(3-methylbutyl) isomer were further tested for their in vivo activity using the elevated plus maze (EPM), which measures locomotive effects (total arm entries) and anxiolytic effects (open arm entries and % time in the open arms). One mg/kg of CP55,940 or 4-fluoro MDMB-BINACA reduced total arm entries compared to vehicle or THC treatment (Fig. 12a–c); whereas THC or JWH 018 2′-napthyl-N-(3-methylbutyl) isomer treatment increased open arm entry and did not change total arm entries (Fig. 12a–c). Mice treated with 4-fluoro MDMB-BINACA spent approximately the same amount of time in the open arm as vehicle-treated mice; but CP55,940- and 4-fluoro MDMB-BINACA-treated mice spent the majority of their time in the central quadrant immobile (Fig. 12d–f). In contrast, THC or JWH 018 2′-napthyl-N-(3-methylbutyl) isomer treatment was associated with a greater amount of time spent in the open arms, indicative of an anxiolytic effect (Fig. 12d–f). Therefore, these two SCRAs which had opposing biases for and against βarrestin2 relative to G protein signaling also differed in their in vivo activity such that βarrestin2 bias was associated with greater cataleptic and hypothermic activity, and G protein bias was associated with greater anxiolytic activity.

## Discussion

In general, we observed that most SCRAs tested behaved as CB1R and CB2R full agonists in the cAMP inhibition assay. However, many did not fully displace [^3^H]CP55,940 from CB1R or CB2R in the competition binding assay. A notable exception to this was AB-PINACA N-(2-fluoropentyl) isomer, a partial agonist for the CB1R cAMP inhibition response and a full agonist of βarrestin2 recruitment. SCRAs generally displayed little to no consistent selectivity between CB1R and CB2R. This is likely due to a high degree of shared receptor homology and topology between the CB1R and CB2R orthosteric binding sites ^20,21,24–26^.

In this study, we did not explore the pharmacology of SCRAs at receptors other than CB1R and CB2R. Naturally-occurring phytocannabinoids such as CBD and THC have affinity for the transient receptor potential vanilloid 1 (TRPV1) and transient receptor potential ankyrin 1 (TRPA1) Ca^2+^ channels, orphan GPCRs, and serotonin receptors, among others ^2^. Similarly, the SCRA WIN55,212-2 has been shown to desensitize TRPV1 and TRPA1 to produce analgesia ^27^. Although it is not known whether the SCRAs studied here are ligands for these other ‘non-canonical’ cannabinoid receptors, modulation of these receptors’ activity by SCRAs could affect nociception, inflammation, behaviour, mood, and appetite ^27^ and therefore explain some of their in vivo activity beyond CB1R and CB2R.

A select few of the SCRAs assayed here produced potent and supramaximal responses in the βarrestin2 recruitment assay at both CB1R and CB2R. Specifically, SCRAs with indazole cores rather than indole cores and halogen-substituted butyl or pentyl groups, such as 4-fluoro MDMD-BINACA, were the most-potent and most-efficacious agonists of βarrestin2 recruitment as CB1R and CB2R. Inclusion of an indole core rather than an indazole core (e.g. 5-fluoro MPP-PICA) or a halogen-substituted benzene rather than butyl or pentyl groups (e.g. meta and ortho AB-FUBINACA) abolished βarrestin2 activity; whereas moving the halogen from the terminal carbon toward the indazole core (e.g. AB-PINACA N-(2-fluoropentyl) isomer and 3-chloro AB PINACA) only reduced βarrestin2 activity. Cannaert et al. ^28^ recently reported similar βarrestin2 activity for 5-fluoro MPP-PICA and 4-fluoro MDMB-BINACA, as well as related SCRAs having indazole cores and fluoro-substituted butyl and pentyl groups. Similar studies have shown a consistent SAR between SCRA potency and efficacy and the incorporation of indazole cores and terminal halogen substitution to butyl and pentyl chains ^15,28–30^. Further, the indazole core has been shown to have a stabilizing effect on the “twin toggle switch” that affords CB1R-Gα_i_ interactions ^17^. It may be that the stabilization of the “twin toggle” in combination with the increased affinity provided by halogen-substituted butyl or pentyl groups promotes sustained receptor activation and therefore, βarrestin2 recruitment ^17^. Previous work has found that many SCRAs structurally and functionally related to the subset studied here display ligand bias and functional selectivity for βarrestin2 recruitment relative to the inhibition of cAMP ^14–19,31–34^. Our data extend previous findings to suggest that these structural elements specifically confer βarrestin2 bias because these structural differences did not dramatically change potency and efficacy in the cAMP assay.

Building from our cell culture data, we observe that the majority of in silico interactions made between SCRAs and either CB1R or CB2R are highly similar, with key homologous residues such as Ser383^7.39^ (CB1R)/Ser285^7.39^ (CB2R) and His178^2.65^ (CB1R)/His95^2.65^ (CB2R) present in our models and crystal structures of these receptors bound to SCRAs ^20,21,24,26^. The binding pocket’s shape and position are retained and consistent for SCRAs at both CB1R and CB2R, with slight differences in amino acid interactions observed between cAMP-biased and βarrestin2-biased compounds (Tables 2 and 3). Perhaps most-intriguing is the observation that interaction energies differed depending on the G protein’s inclusion or exclusion in our model. The highly βarrestin2-biased ligand 4-fluoro MDMB-BINACA incurred a 10.56 kcal/mol binding energy penalty in the presence of the G protein (Table 4). In contrast, the G protein-biased JWH 018 2′-naphthyl-N-(3-methylbutyl) isomer gained 3.78 kcal/mol in interaction energy in the presence of the G protein (Table 4). Although these static in silico models cannot recapitulate the dynamic interactions occurring between ligand and receptor in vivo, they do add support to the growing body of evidence for unique binding interactions for βarrestin2-biased ligands at the cannabinoid receptors ^29,30^.

How do the kinetics of binding differ between the SCRAs studied here and the more benign partial CB1R and CB2R agonists such as THC, AEA, and 2-AG? Data presented here suggest that despite many SCRAs being full agonists, they incompletely displace CP55,940 in vitro. In silico modelling of CB1R and CB2R indicates that while SCRAs occupy a similar site in the cannabinoid receptors, specific amino acid residue interactions differ from ligand-to-ligand. SCRAs such as the indole- and indazole-core ligands largely focused on here appear to interact with the cannabinoid receptors via unique receptor confirmations that differentially affect receptor-mediated signaling. Specific SCRAs with isothiocyanate substitutions have been designed to covalently bind CB1R (e.g. AM6538 and AM841) ^25,26,35^, but no evidence to date indicates that other SCRAs covalently bind the cannabinoid receptors ^14–19,31–34^. The unique binding modes of SCRAs may dramatically increase on-rate or slow off-rate of ligands from the receptors and consequently alter the pharmacodynamics of SCRAs ^34,36^, such as the βarrestin2 bias and catalepsy observed with low concentrations of 4-fluoro MDMB-BINACA. This work and previous studies point to specific amino acids within CB1R and CB2R that may dictate ligand bias and kinetics, such as Ser383^7.39^ and His178^2.65^ (CB1R) ^20,21,24–26^. Planned future in silico and in vitro studies will explore the on- and off-rate binding kinetics of SCRAs with mutagenized and wild-type receptors.

The translation of observed in vitro bias to in vivo effects remains a key limitation in the study of biased agonism ^37,38^. Here, we observed that the highly βarrestin2-biased ligand 4-fluoro MDMB-BINACA was the most potent and efficacious agonist of catalepsy and hypothermia among the cannabinoids tested. In contrast, the G protein-biased JWH 018 2′-naphthyl-N-(3-methylbutyl) isomer was less efficacious in the catalepsy assay, did not produce hypothermia, and reduced anxiety-like behaviours in the EPM. Two related compounds, MDMB-FUBINACA and 5-fluoro AMB, have similarly been shown to produce profound hypothermic responses in Long-Evans rats at these doses ^15^. Structural analogues of these ligands, such as XLR-11 ^39^, 5-fluoro CUMYL-P7AICA ^16^, CUMYL-4CN-BINACA ^40^, and AB-CHMINACA, and AB-PINACA ^41^ have shown similar cannabimimetic effects in rodent models—including profound hypothermia and pro-convulsant effects. Here, in vitro and in silico experiments utilized human CB1R and CB2R, whereas in vivo experiments were conducted in mice, which represents a limitation of our study. Subtle differences in CB1R and CB2R-dependent βarrestin recruitment have been documented between species ^42^; however, ligand affinity and activity, and sequence similarity have been shown to be highly similar between human and mouse CB1R and CB2R ^11,18,19,22^. Another possible reason for varying activity in vivo could be differences in non-specific plasma protein binding among SCRAs. However, pharmacokinetic analyses of AM 2201 and JWH-018 N-(5-hydroxypentyl) in rats show that these compounds have similar plasma protein binding, rates and extents of metabolism, and their efficacy correlates with plasma concentrations ^43^. Similarly, a recent meta-analysis in humans found no significant difference in free plasma serum concentrations of 65 SCRAs across 117 reports published between 2009 and 2020 [reviewed in^44^]. As a limitation of our data, equivalent mouse pharmacokinetic data do not exist for 4-fluoro MDMB-BINACA and JWH 018 2′-naphthyl-N-(3-methylbutyl) and such data are beyond the scope of the present study. However, existing data presented here and elsewhere indicate that differences in SCRAs activity are pharmacodynamic rather than pharmacokinetic ^45–47^. Although previous studies have not correlated the βarrestin2 bias of such ligands with specific in vivo effects, it is possible that βarrestin2-biased signaling may selectively enhance hypothermic and cataleptic responses relative to other cannabimimetic effects such as anti-nociception. Additional research is required to test this hypothesis directly.

Is βarrestin2 bias responsible for the on-target pharmacodynamic toxicities associated with SCRAs ^39,41,48^? Specifically, are hypothermia and catalepsy the functional repercussions of supramaximal βarrestin2 recruitment via the cannabinoid receptors? Increasing data from other GPCR systems, such as the µ-opioid receptor and serotonin 5HT2A receptor, indicate that undesirable effects of receptor activation may be the result of functional selectivity toward βarrestin2 and away from G protein-mediated signaling cascades ^37,38,49^. In the case of the µ-opioid receptor, respiratory depression, constipation, and possibly addictive potential, appear to be βarrestin2-dependent ^38^. For 5HT2A, hallucinogenic effects of lysergic acid diethylamide (LSD)—as modelled in rodents—may be βarrestin2-dependent ^49^. As with the second question posed above, understanding the molecular mediators of the SCRAs’ toxicities may aid in limiting their harm. Our work has previously shown that βarrestin-biased ligands produce CB1R internalization, overall CB1R downregulation, and reduced cell viability in cultured neurons ^11,18^. Therefore, it is critical that we determine whether supra-physiological βarrestin2 recruitment by some SCRAs ^15,16,40^ augments CB1R internalization and downregulation, and reduces cell viability ^11,18^. Whether βarrestin2 bias dictates the differential signaling of SCRAs in vivo in both acute and chronic treatment paradigms must also be determined. In human case studies, illicit SCRA use confirmed by blood analysis is linked to delirium, agitation, lethargy, immobility, and tachycardia and associated cardiovascular events [reviewed in ^44,50^]. These clinical data include compounds we observed to be G protein-biased (e.g. AM 2232) and βarrestin2-biased (e.g. 5-fluoro NPB-22), but the relationship between SCRA potency and toxicity in humans is uncharacterized at this time. We observed profound immobility with the βarrestin2-biased SCRA 4-fluoro MDMB-BINACA, but did not assess cardiovascular physiology in mice. Understanding such adverse effects, and differentiating them from the partial agonism of plant-derived phytocannabinoids such as THC, may aid in our understanding of the unique toxicities associated with SCRAs ^39,41^. Future mouse studies will be conducted utilizing wild-type and βarrestin2 knockout mice to determine the consequence of SCRA βarrestin2 bias on neuronal viability, desensitization, cognition, movement, and cardiac function; as has been done for both the study of novel cannabinoids and for understanding the functional consequences of ligand bias at other receptor systems in the past ^37^.

The cannabinoid receptors are capable of accommodating a wide array of structurally diverse agonists. This study describes some of the SAR of SCRAs in vitro, in silico, and in vivo at CB1R and CB2R. Future extensions of this research will need to explore a greater variety of cannabinoid-targeted receptors, such as those mentioned above. As data on the SCRAs continue to accumulate, we hope to improve our ability to predict the pharmacodynamic profiles of SCRAs based on their chemical structures.

## Methods

### Compounds

All compounds were purchased from Cayman Chemicals (Ann Arbor, MI) with the exception of ∆^9^-tetrahydrocannabinol, which was purchased from Toronto Research Chemicals (Toronto, ON). [^3^H]CP55,940 (174.6 Ci/mmol) was obtained from PerkinElmer (Guelph, ON). All other reagents were obtained from Sigma-Aldrich (Oakville, ON) unless specifically noted. Compounds were dissolved in DMSO (final concentration of 0.1% in assay media for all assays) and added directly to the media at the concentrations and times indicated.

### Cell culture

Chinese hamster ovary (CHO)-K1 cells, either untransfected for use as controls in saturation binding studies or stably expressing human CB1R or CB2R, were cultured as described in earlier studies from our group ^19^. Briefly, cells were maintained at 37 °C, 5% CO_2_ in F-12/DMEM containing 1 mM l-glutamine, 10% FBS, and 1% Pen/Strep as well as hygromycin B (300 µg/mL) and G418 (600 µg/mL) for CHO-K1 hCB1R cells or G418 (400 µg/mL) for CHO-K1 hCB2R cells ^19,51^. In the case of membrane collection for radioligand binding, cells were scraped from flasks, centrifuged, and frozen at − 80 °C until required. HitHunter (cAMP) and PathHunter (βarrestin2) CHO-K1 cells stably-expressing hCB1R or hCB2R from DiscoveRx (Eurofins, Fremont, CA) were maintained at 37 °C, 5% CO_2_ in F-12 DMEM containing 10% FBS and 1% penicillin–streptomycin with 800 µg/mL geneticin (HitHunter) or 800 µg/mL G418 and 300 µg/mL hygromycin B (PathHunter), as described previously ^19^.

### CHO cell membrane preparation and radioligand displacement assay

Cells were thawed, diluted in Tris buffer (50 mM Tris–HCl and 50 mM Tris–base) and homogenized in a 1 mL hand-held homogenizer ^19,51^. Untransfected, hCB1R, and hCB2R CHO-K1 cell membranes were collected by cavitation in a pressure cell, and sedimented by ultracentrifugation, as originally described in Bolognini et al. ^41^. Pellets were resuspended in TME buffer (50 mM Tris–HCl, 5 mM MgCl_2_, 1 mM EDTA, pH 7.4) and protein concentration was measured via the Bradford method (Bio-Rad Laboratories, Mississauga, ON).

Assays have been described in detail previously and are summarized here ^19,52^. Saturation binding experiments were conducted with 0.1–100 nM [^3^H]CP55,940 and Tris binding buffer (50 mM Tris–HCl, 50 mM Tris–base, 0.1% BSA, pH 7.4, 2 mL) ^19,52^. Saturation binding experiments utilized CHO-K1 cells expressing either hCB1R or hCB2R (total bound) and untransfected CHO-K1 cells (non-specific bound). Specific binding curves were calculated as the difference between total and non-specific for each concentration of [^3^H]CP55,940 used and fit to a one site total binding non-linear regression (GraphPad, Prism, v. 9.0) (Supplementary Fig. 1). Competition binding experiments were conducted with 0.7 nM [^3^H]CP55,940, SCRAs as described, and Tris binding buffer (50 mM Tris–HCl, 50 mM Tris–base, 0.1% BSA, pH 7.4, 2 mL) ^19,52^. Radioligand binding began with the addition of CHO-K1 cell membranes (50 µg protein per sample). Assays were performed for 120 min at 37 °C and stopped by the addition of ice-cold Tris binding buffer, followed by vacuum filtration using a 24-well sampling manifold (Brandel Cell Harvester; Brandel Inc, Gaithersburg, MD, USA). Brandel GF/B filter paper was soaked with wash buffer at 4 °C for at least 24 h. Each filter paper was washed six times with a 1.2 mL aliquots of Tris-binding buffer, then air-dried overnight and submerged in 5 mL of scintillation fluid (Ultima Gold XR, PerkinElmer). Liquid scintillation spectrometry was used to quantify radioactivity. For competition binding experiments, specific binding was equal to the difference in radioactivity with or without 1 µM unlabelled CP55,940.

### HitHunter cAMP assay

Quantification of FSK-stimulated cAMP accumulation using the DiscoveRx HitHunter assay has been described previously and is summarized here ^19^. Cells (20,000 cells/well in low-volume 96 well plates) were incubated overnight in Opti-MEM containing 1% FBS at 37 °C and 5% CO_2_. Following this, Opti-MEM media was removed and replaced with cell assay buffer (DiscoveRx) and cells were co-treated at 37 °C with 10 µM FSK and ligands for 90 min. cAMP antibody solution and cAMP working detection solutions were added to cells (DiscoveRx), and cells were incubated for 60 min at room temperature. cAMP solution A (DiscoveRx) was added and cells were incubated for an additional 60 min at room temperature before chemiluminescence was measured on a Cytation5 plate reader (top read, gain 200, integration time 10,000 ms).

### PathHunter βarrestin2 assay

Quantification of βarrestin2 recruitment using the DiscoveRx PathHunter assay has been described previously and is summarized here ^19^. Briefly, cells (20,000 cells/well in low-volume 96 well plates) were incubated overnight in Opti-MEM containing 1% FBS at 37 °C and 5% CO_2_. Cells were co-treated with ligands for 90 min at 37 °C. Detection solution was added to cells (DiscoveRx), and cells were incubated for 60 min at room temperature. Chemiluminescence was measured on a Cytation5 plate reader (top read, gain 200, integration time 10,000 ms).

### Molecular docking

The published cryo-EM structure PDB: 6N4B ^20^ was used for the molecular modelling of CB1R. This cryo-EM structure contains the agonist MDMB-FUBINACA bound to CB1R-Gα_i_. CB2R modelling utilized PBD: 6PT0 ^21^ which contains the agonist WIN 55,212-2 bound to CB2R-Gα_i_. Cannabinoids were modelled docking to GPCRs both with the Gα_i_ present to simulate the G protein-bound state or with the Gα_i_ removed to simulate the G protein-absent state that may be more conducive to βarrestin2 coupling. Each of the cannabinoids was built and optimized in Spartan ’18 Parallel Suite ^53^ and docked via induced fit docking in Schrödinger, as described previously ^53–55^. To reduce atom clashing, ligand-receptor complexes were minimized using the OPLS3 force field in Prime ^54^. Prime MM-GBSA was used to calculate the interaction energy between each ligand and the receptor ^54^.

### Animals

Adult male C57BL/6 mice aged 6–10 weeks (mean weight 24 ± 0.5 g) were purchased from Charles River Labs (Senneville, QC). Animals were group housed (three per cage) with ad libitum access to food, water, and environmental enrichment and maintained on a 12 h light/dark cycle. Mice were randomly assigned to receive *i.p.* injections of vehicle (1:1:18 ethanol:emulphor:saline) or 0.1–10 mg/kg SCRA (*n* ≥ 4 per group)*.* All protocols have been described previously ^19^ and were in accordance with the guidelines detailed by the Canadian Council on Animal Care ^56^ and approved by the Animal Research Ethics Board and the Scientific Merit Review Committee for Animal Behaviour at the University of Saskatchewan. The following steps were taken to ensure our study was in keeping with the ARRIVE guidelines: (1) power analyses were conducted to determine the minimum number of animals required for the study; (2) animals were purchased—rather than bred—to limit animal waste; and (3) all assessments of animal behaviour were made by individuals blinded to the treatment group ^56,57^.

Modified tetrad testing (catalepsy, body temperature, tail flick assay) was conducted according to previously described methods, which are summarized briefly here ^19^. Time points for testing were chosen based on previously published findings ^19,22,23^ and a time course experiment conducted with 10 mg/kg CP55,940 (Supplementary Fig. 3). Catalepsy was assessed in the ring holding assay 5 min following injection with mouse forepaws clasped to a 5 mm ring positioned 5 cm above the surface of the testing space. The length of time the ring was held was recorded (s) to a maximum time of 60 s (i.e. MPE = 60 s). Internal body temperature was measured 15 min after injection. Anti-nociception was determined by assessing tail flick latency 20 min after injection. Mouse tails were placed ~ 1 cm into 52 ± 2 °C water and the time until the animal removed its tail was recorded as tail flick latency (s) up to 20 s (i.e. MPE = 20 s).

EPM was conducted according to methods adapted from Marks et al. ^58^. The EPM was built from a plywood base painted blue with blue corrugated plastic floor and walls. The floor of the maze was composed of perpendicular interlocking arms (110 cm long × 10 cm wide) with two arms having 45 cm walls (closed arms) and two arms having no walls and a 0.5 cm edge (open arms), all elevated 45 cm from the ground ^58^. Trials began with the mouse in the central zone facing an open arm. Trials lasted 5 min and began 30 min after SCRA treatment. An arm entry was considered to be all four limbs leaving the central zone. Arm entries and the percent time spent in open versus closed arms were recorded by an observer blinded to the treatment group. Less time in the open arms and more time in the closed arms was used as a model of anxiogenic behaviour ^58^.

### Statistical analyses

[^3^H]CP55,940 radioligand saturation binding data are provided as raw counts per minute (cpm) bound and fit to a one site total binding non-linear gression (GraphPad) (Supplementary Fig. 1). [^3^H]CP55,940 radioligand competition binding data are provided as % change from maximal ^3^H bound (i.e. 100%). Data for HitHunter cAMP and PathHunter βarrestin2 data are shown as % of maximal CP55,940 response (i.e. 100%). Estimates of *K*_i_, EC_50_, *E*_min_, and *E*_max_ were determined using non-linear regression with variable slope (four parameters) (GraphPad, Prism, v. 8.0). The operational model of Black and Leff ^59^ was used to estimate bias (∆∆LogR) with CP55,940 as the reference agonist ^11,18,19^. One-way analysis of variance (ANOVA), followed by Tukey’s post-hoc test, was used for statistical analyses (p < 0.05 determined to be significant); and Bartlett’s test was used to confirm homogeneity of variance (GraphPad). Values are presented as the mean ± the standard error of the mean (SEM) or 95% confidence interval (CI), as indicated in tables and figure legends.

## Supplementary Information

Supplementary Figures.
