# Supplementary material for: Assessment of select synthetic cannabinoid receptor agonist bias and selectivity between the type 1 and type 2 cannabinoid receptor
Source: Sci Rep. 2021 May 19;11:10611. doi: 10.1038/s41598-021-90167-w (PMC8134483; doi:10.1038/s41598-021-90167-w)
Supplement: Supplementary file 1 — Supplementary Figures. [file 41598_2021_90167_MOESM1_ESM.docx]

**Supplementary Material: Assessment of select synthetic cannabinoid receptor agonist bias and selectivity between the type 1 and type 2 cannabinoid receptor.**

Ayat Zagzoog^1^, Asher L Brandt^1^, Tallan Black^1^, Eunhyun D Kim^1^, Riley Burkart^1^, Mikin Patel^2^, Zhiyun Jin^2^, Maria Nikolaeva^2^, Robert B Laprairie^1,3*^

**Supplementary Figure 1. [^3^H]CP55,940 saturation binding to CB1R and CB2R.** Binding of 0.1 – 100 nM [^3^H]CP55,940 to membranes obtained from CHO cells stably-expressing hCB1R **(a)** or hCB2R **(b)**. Specific binding was estimated by subtracting non-specific binding values – obtained from the membranes of untransfected CHO-K1 cells – from the total binding values in either hCB1R CHO-K1 or hCB2R CHO-K1 cells, respectively. Data were fit to a one site total binding non-linear regression using GraphPad v. 9. Data are mean ± S.E.M., or mean with 95% C.I. (*K*_d_), *n* = 3 independent experiments performed in triplicate.

**Supplementary Figure 2. Cannabinoid receptor-dependent inhibition of FSK-stimulated cAMP.** CHO cells stably-expressing hCB1R (**a**) or hCB2R (**b**) were treated with vehicle (10% DMSO in PBS), 10 µM FSK, and 10 μM CP55,940 for 90 min as indicated above and cAMP accumulation was measured. Here, cAMP accumulation is expressed as untransformed relative light units from the experiment. In subsequent data analyses, data are expressed as % inhibition relative to the maximal CP55,940 response as illustrated above by dotted lines. Data were analyzed in GraphPad v. 8. Data are mean ± S.E.M. *n* = 6 independent experiments shown with circles (performed in triplicate).

**Supplementary Figure 3. *In vivo* time course.** Male C57Bl/6 mice were treated with 10 mg/kg  *i.p.* CP55,940 and assessed for their catalepsy (ring holding, **a**), body temperature (rectal thermometer, **b**), and nociceptive response (warm water tail flick latency, **c**). Based on these data, peak effect times for physiological assessments were chosen to be (**a**) catalepsy, 5 min; (**b**) body temperature, 15 min; and (**c**) nociception, 20 min for *in vivo* assessments described in figure 11.  Data are mean ± S.E.M., *n* = 4 animals.
